# Rottlerin-mediated inhibition of *Toxoplasma gondii* growth in BeWo trophoblast-like cells

**DOI:** 10.1038/s41598-017-01525-6

**Published:** 2017-04-28

**Authors:** Francesca Ietta, Emanuela Maioli, Elena Daveri, Juliana Gonzaga Oliveira, Rafaela José da Silva, Roberta Romagnoli, Laura Cresti, Anna Maria Avanzati, Luana Paulesu, Bellisa de Freitas Barbosa, Angelica de Oliveira Gomes, José Roberto Mineo, Eloisa Amália Vieira Ferro

**Affiliations:** 1 Department of Life Sciences, University of Siena, Via A. Moro 4, 53100 Siena, Italy; 2 Laboratory of Immunophysiology of Reproduction, Institute of Biomedical Sciences, Federal University of Uberlândia, Av. Pará 1720, 38405320 Uberlândia, Brazil; 3 Laboratory of Immunoparasitology, Institute of Biomedical Sciences, Federal University of Uberlândia, Av. Pará 1720, 38405320 Uberlândia, Brazil

## Abstract

Autophagy is a crucial and physiological process for cell survival from yeast to mammals, including protozoan parasites. *Toxoplasma gondii*, an intracellular parasite, typically exploits autophagic machinery of host cell; however host cell upregulates autophagy to combat the infection. Herein we tested the efficacy of Rottlerin, a natural polyphenol with autophagic promoting properties, against *Toxoplasma* infection on the chorioncarcinoma-derived cell line BeWo. We found that Rottlerin, at sub-toxic doses, induced morphological and biochemical alterations associated with autophagy and decreased *Toxoplasma* growth in infected cells. Although autophagy was synergically promoted by *Toxoplasma* infection in combination with Rottlerin treatment, the use of the autophagy inhibitor chloroquine revealed that Rottlerin anti-parasitic effect was largely autophagy-independent and likely mediated by the converging inhibitory effect of Rottlerin and *Toxoplasma* in host protein translation, mediated by mTOR inhibition and eIF2α phosphorylation. Both events, which on one hand could explain the additive effect on autophagy induction, on the other hand led to inhibition of protein synthesis, thereby depriving *Toxoplasma* of metabolically essential components for multiplication. We suggest that modulation of the competition between pathogen requirement and host cell defense might be an attractive, novel therapeutic approach against *Toxoplasma* infection and encourage the development of Rottlerin-based new therapeutic formulations.

## Introduction

Autophagy is an evolutionary conserved process of cellular degradation that is crucial for cell survival, differentiation and development from yeast to mammals, including protozoan parasites^1^. The autophagic process is controlled by several AuTophagy Genes (ATGs) through a multi-step mechanism that includes induction, cargo recognition, autophagosome formation/fusion with lysosomes and cargo digestion followed by release of the degradation products^2^. Autophagy is constitutively active on a basal level helping to sustain cellular functions, but it can be strongly induced in response to a multitude of stimuli, such as nutrients deprivation, hypoxia and pathogen infection (xenophagy), to name but a few. Although this process can be seen as a survival mechanism to deal with nutrient limitation, the prolonged and intense activation of autophagy can be lethal, because of self-degradation of essential cellular components^3^. Thus, autophagy can be a cell survival mechanism in certain circumstances, but a mediator of cell death in others. Autophagy is indeed a type of programmed cell death (Type II death) that has a distinct progression from that of the Type I apoptotic death and is particularly relevant in those cells where the apoptotic machinery is compromised, such as in certain cancer cells^4, 5^ or in protozoa, where a typical apoptosis has not been universally demonstrated^6^.

*Toxoplasma gondii* is an intracellular protozoan parasite that infects virtually any type of nucleated cells from a wide range of warm-blooded vertebrates, including humans. The prevalence of toxoplasmosis varies greatly around the world, it has been estimated that approximately 30% of human population worldwide is chronically infected with *T. gondii* ^7^. Primary infection in adults results in mild or nonspecific symptoms^8^. However in immunocompromised individuals, primary infection or reactivation of chronic infection can cause fatal toxoplasmicencephalitis, myocarditis and pneumonitis^9^. In addition, toxoplasmosis is associated with severe congenital defects when primary infection is acquired during pregnancy, especially during the first trimester of pregnancy^8^.

Moreover, because *Toxoplasma* is an obligated intracellular pathogen, the autophagic process switched on by the host cell (xenophagy) might be determinant for the parasite fate. In fact, while *T. gondii* typically exploits the autophagic machinery of the host cell to its own advantage^10^, the host cell upregulates autophagy to combat the infection. It follows that there might be a threshold, non-deadly for the host cell, beyond which autophagy leads to parasite death. Modulation of the competition between pathogen requirement and host cell defense could be therefore an attractive and novel therapeutic approach.

Following this hypothesis, in the current study, we tried to perform a precise autophagy-targeted approach, based on the known autophagy promoting effects of Rottlerin^4, 10, 11^.

As recently reported, Rottlerin induced autophagy through inhibition of mTORC1, a negative regulator of autophagy^5^. Importantly, mTORC1 is also a key player in the control of protein synthesis, which, conversely, is stimulated. Indeed, the initiation step of mRNA translation is commenced by the binding of the eukaryotic translation initiation factor 4 F (eIF4F) complex to the cap-structure of mRNA. eIF4E is regulated by 4E-BP, whose phosphorylation by mTORC1 causes the release of free eIF4E that will then initiate protein synthesis. Thus, mTORC1 inhibition results in both autophagy induction and translational arrest^12^.

Rottlerin is a natural polyphenol isolated from *Mallotus philippinensis*, an ancient traditional Indian medicinal plant in Ayurvedic Medicine System, mainly known for its anthelminthic activity^13, 14^. Rottlerin, however, has potential beneficial applications in a variety of other ailments, being an antioxidant^15^, anti-inflammatory^16, 17^, antiallergic^17^, antibacterial^18, 19^ and anticancer compound^20^. Rottlerin is a very versatile substance that has been used for decades as a selective PKCδ inhibitor^21^ though it is now clear that Rottlerin also inhibits other enzymes^22^ and acts as a mitochondrial uncoupler^23^.

Given the pleiotropic properties of Rottlerin, in particular its autophagy inducing ability and the importance of *T. gondii* infection during pregnancy^8^, the aim of the current study was to test the efficacy of Rottlerin against the parasite, in the trophoblast-like cell line BeWo, the most extensively used cellular *in vitro* model for villous trophoblast studies.

Autophagy is physiologically involved in normal placentation^24^ and recent studies suggest that autophagy in trophoblasts has primarily an adaptive role^25^. An increase in autophagy flux is indeed associated with cytoprotective mechanisms of the trophoblast cells against micro environmental challenges^24, 26^. Hence, autophagy induction is expected to be well tolerated by BeWo cells.

However, because of the several, potentially harmful, Rottlerin effects, the drug needs to be used with caution. Then, the main challenge of this work was to individuate the optimal dose that is not toxic for the host cell, but is lethal for the parasite.

## Results

### BeWo cells viability

**Figure 1:**
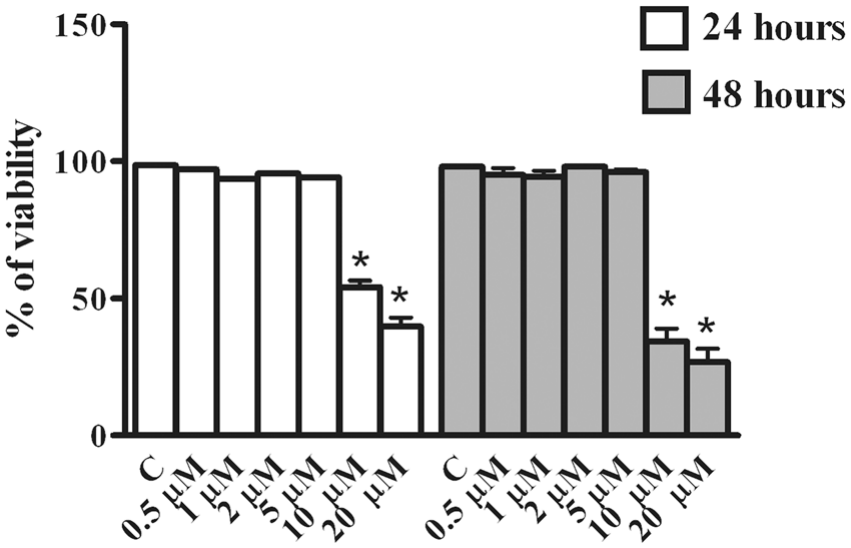
Effect of Rottlerin treatment on BeWo cells viability. BeWo cells were treated for 24 and 48 hours with increasing concentrations of Rottlerin (0.5 µM–20 µM) and cell viability was evaluated by direct cell counting (Trypan Blue). Data are expressed as a percentage of control (vehicle-treated)cultures and reported as mean ± SEM. The results represent four independent experiments performed in quadruplicate for each data point. *p < 0.05.

The effect of Rottlerin on viability of BeWo cells was evaluated by Trypan Blue exclusion assay. As reported in Fig. 1, no difference in cell viability with respect to control (vehicle-treated cultures), was observed when BeWo cells were treated with Rottlerin at concentrations from 0.5 to 5 µM, neither at 24 nor at 48 hours. On the other hand, significant decrease of cell viability was observed at the concentrations of 10 and 20 µM at both 24 and 48 hours (Fig. 1). Specifically, cell viability was reduced to around 60% after 24 hours of 10 µM Rottlerin treatments and to less than 50% after 48 hours. The decrease was more marked with 20 µM Rottlerin, being cell viability reduced to less than 50% already after 24 hours of incubation, and to around 30% after 48 hours. Based on these data we performed subsequent experiments using Rottlerin at concentrations of 0.5, 1, 2 and 5 µM.

### Rottlerin decreases *T. gondii* growth in BeWo cells

**Figure 2:**
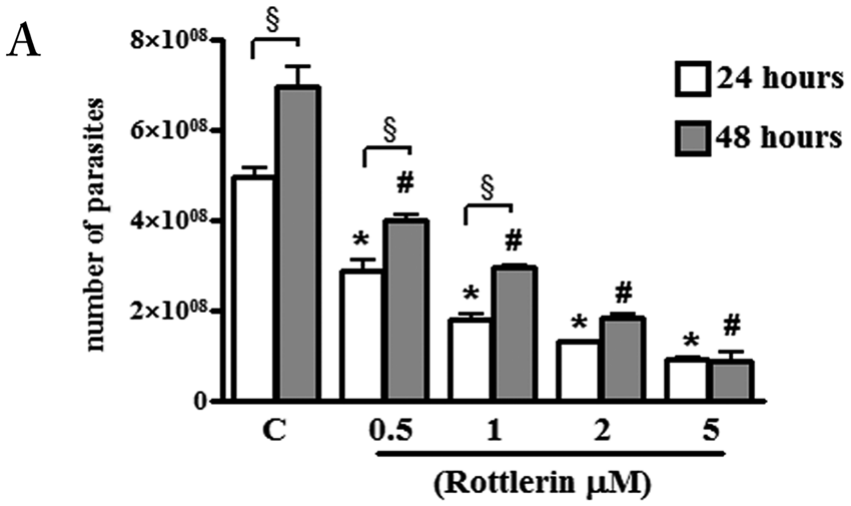
Effect of Rottlerin treatment on *T. gondii* proliferation. BeWo cells were infected with *T. gondii* (host cell: tachyzoites, 1:5) for 3 hours then treated with increasing concentrations of Rottlerin (0.5 µM–5 µM). *T. gondii* proliferation was evaluated after 24 and 48 hours by a colorimetric microtiter assay using β-galactosidase-expressing tachyzoites. Data were expressed as mean ± SEM of the number tachyzoites calculated in relation to a reference curve and are representative of three independent experiments performed in triplicate. *p < 0.0001: Rottlerin -treated *vs* control-vehicle cultures at 24 hours; ^#^p < 0.01: Rottlerin -treated *vs* control-vehicle cultures at 48 hours; ^§^p < 0.05: Rottlerin-treated cultures at 24 hours *vs* 48 hours.

The effect of Rottlerin on parasite growth was evaluated in BeWo cells infected for 3 hours and then treated with the compound for 24 and 48 hours. Our results showed that increasing concentrations of Rottlerin resulted in significant inhibition of tachyzoites growth from around 50% (0.5 µM Rottlerin) to 85% (5 µM Rottlerin) as respect to vehicle-treated cell cultures after 24 as well as after 48 hours cultures (Fig. 2). Interestingly, while a significant increase was observed in tachyzoites number from 24 to 48 hours in 0.5, 1 and 2 µM Rottlerin-treated cultures, no evidence of parasite growth was found in the same time interval at the dose of 5 µM (Fig. 2). Therefore, this dose was chosen for the subsequent experiments. The indices of infection and parasite replication were determined in BeWo after 24 h of infection with or without Rottlerin treatment by a direct observation under a light microscope (see Supplementary Figure S1). Results showed a significant decrease of infected cells as well as a significant decrease in the number of parasites per cells (Supplementary Figure S1).

### *Toxoplasma gondii* and Rottlerin are able to induce morphological and biochemical alterations associated with autophagy in BeWo Cells

Due to the well-known properties of Rottlerin as a pro-autophagic drug, we performed morphological and biochemical analysis to investigate on the significant reduction of parasites number observed in the current study.

**Figure 3:**
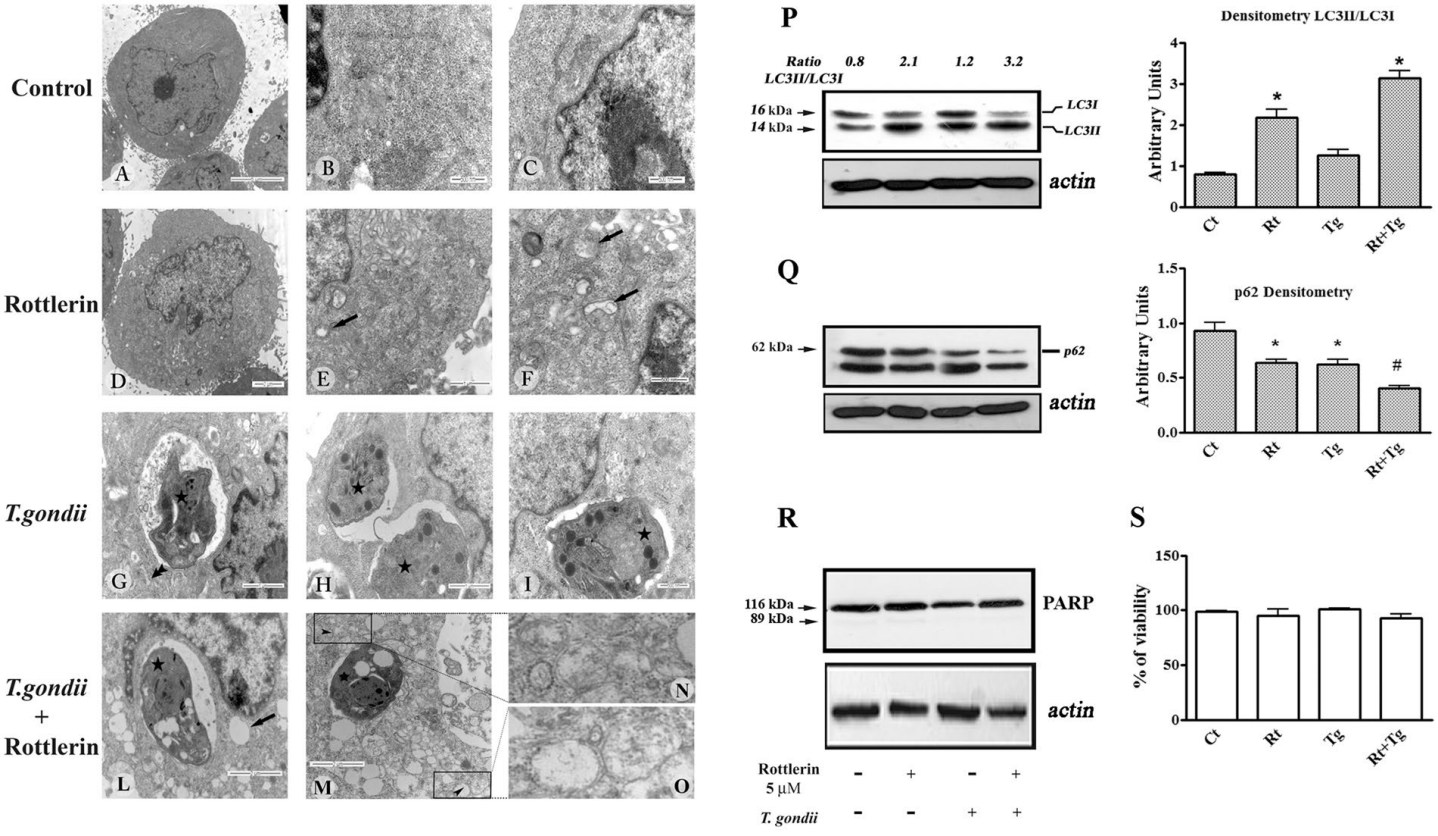
*T. gondii* and Rottlerin induce morphological and biochemical alterations associated with autophagy in BeWo Cells. Left panel: control-vehicle treated cultures (**A**,**B** and **C**); 5 µM Rottlerin-treated cultures (**D**,**E** and **F**); *T. gondii* infected cultures (**G**,**H** and **I**); *T. gondii* infected and 5 µM Rottlerin treated cultured (**L**,**M**,**N** and **O**). Arrows: cytoplasmic vacuoles; star: *T. gondii*; double arrow head: expansion of the rough endoplasmic reticulum; arrow head: double membraned vacuoles (**P** and **Q**): representative western blot and densitometric analysis performed on three independent experiments of LC3I/II (**P**) and p62 (**Q**) proteins in BeWo cells after infection and treatment with 5 µM Rottlerin. *p < 0.05; ^#^p < 0.01. (**R**): representative western blot of three independent experiments of PARP proteins in BeWo cells after infection and treatment with 5 µM Rottlerin. (**S**): viability of BeWo cells after infection and treatment with 5 µM Rottlerin (Rt). (**A**) Bar = 5 µm; (**B**,**C**,**F** and **I**), Bar = 500 nm; (**D**,**L** and **M**), Bar = 2 µm; (**E**,**G**,**H**), Bar = 1 µm.

Electron microscopy (Fig. 3 left panel) reveled that BeWo cells untreated and not infected showed typical morphological characteristics with nuclear decondensed chromatin and evident nucleolus, cytoplasm containing mitochondria, polyribosomes and granular endoplasmic reticulum (Fig. 3A–C). When BeWo cells were treated with 5 µM Rottlerin for 24 hours, morphological changes were observed with the occurrence of cytoplasmic vacuoles and multivesicular bodies (Fig. 3D–F) but no signs of nuclear alterations were visible (Fig. 3D). BeWo cells infected with *T. gondii* showed a slight expansion of the rough endoplasmic reticulum (Fig. 3G–I). Of note, when BeWo cells were infected with *T. gondii* and treated with 5 µM Rottlerin for 24 hours, dramatic morphological alterations, with many cytoplasmic vacuoles as well as numerous multivesicular bodies, were observed (Fig. 3L,M). Of note, the presence of double membraned vacuoles indicates their autophagosomal nature (Fig. 3N and O), which was confirmed by the immunoblotting analysis of cell lysates showing an increase in the lipidated form of LC3 (LC3II) and a reduction/degradation of p62 protein, hallmarks of autophagic flux, both in Rottlerin treated and infected BeWo cells (Fig. 3P and Q). Importantly, as it can be appreciated in Fig. 3Q, the expression of p62 was more markedly reduced and LC3II enhanced when BeWo cells were *T. gondii* infected and then treated with 5 µM Rottlerin.

At the same time, we found that BeWo cell viability was unaffected by the infection and 5 µM Rottlerin treatment or a combination of the two (Fig. 3S). This result, along with the lack of nuclear alterations (Fig. 3D) and PARP cleavage (Fig. 3R), excludes the occurrence of apoptotic cell death at this Rottlerin dose.

### Beclin-1 and mTORC1 involvement in Rottlerin- and Toxoplasma-triggered autophagy

**Figure 4:**
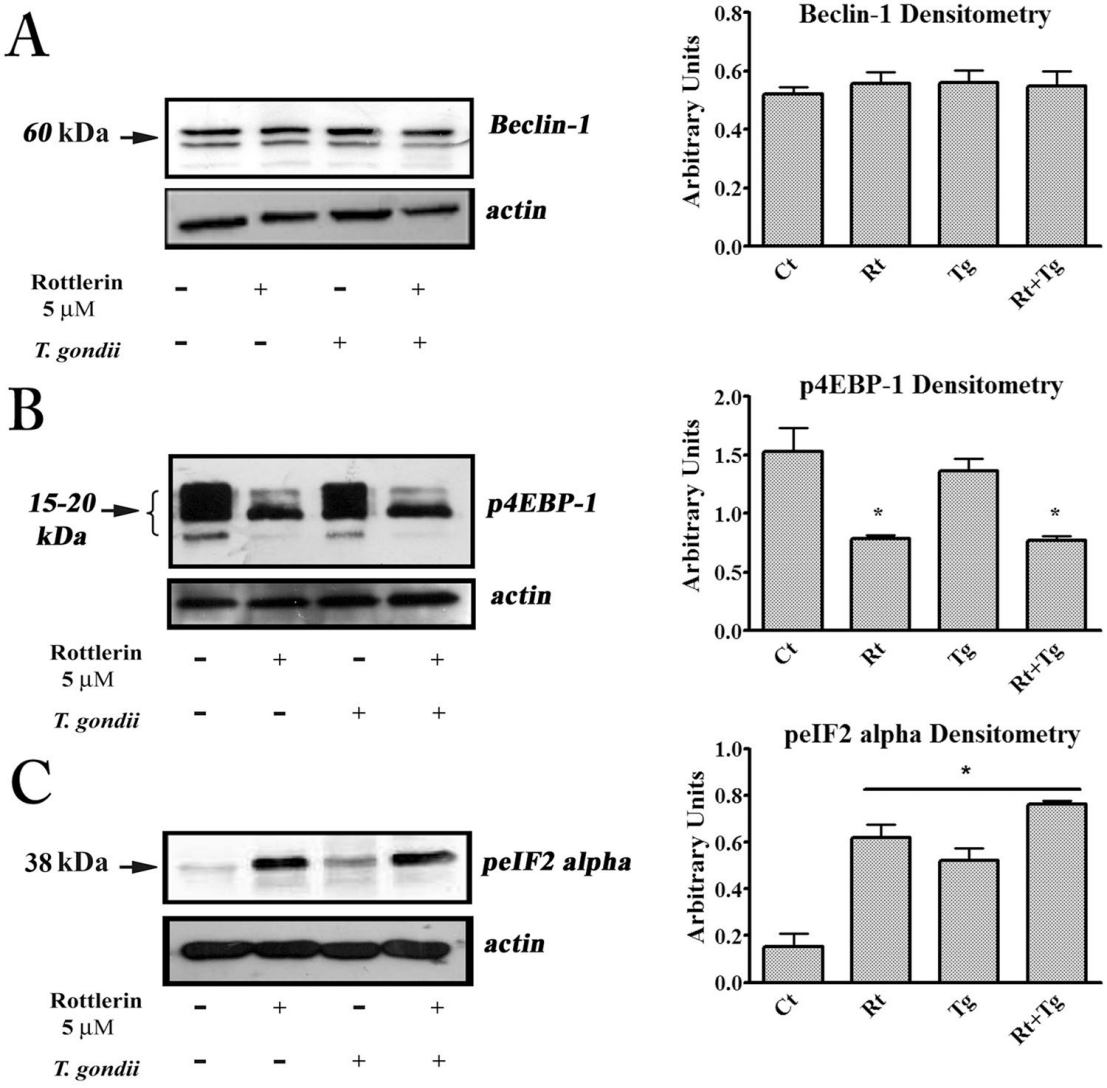
*T. gondii* and Rottlerin engage different autophagic pathways. Representative western blot and densitometric analysis, performed on three independent experiments, of Beclin-1 **(A)**, phosphorylated forms of 4EBP-1 **(B)** and phosphorylated eIF2α (**C**) in BeWo cells after infection and treatment with 5 µM Rottlerin. *p < 0.05.

The two main pathways eventually involved in the autophagy process, Beclin-1, the mammalian orthologue of yeast Atg6/Vps30 with a key role in autophagosome formation^27^ and the mTORC1 cascade^28^ were investigated. As shown in Fig. 4A, no change (induction) of Beclin-1 was observed both in the Rottlerin-treated and in the *T. gondii* -infected cells. Conversely, Rottlerin treatment, but not *T. gondii* infection, decreased the levels of the phosphorylated forms of 4EBP-1 isoforms (Fig. 4B), indicating that, in agreement with previous findings^5^, Rottlerin induced autophagy via inhibition of mTORC1 kinase activity. Given the independence from both Beclin-1 and mTORC1 in *T. gondii*-driven autophagy in the host cell, we presumed the involvement of the endoplasmic reticulum (ER) stress/eIF2α axis as a likely mechanism^29^. The results from Western blotting analysis of BeWo cell lysates after infection, indicated the ER stress-stimulated kinase, PERK, was activated, since eIF2α phosphorylation (a marker of ER stress) increased (Fig. 4C). Moreover, in agreement with the literature^30^, we found that Rottlerin alone was able to trigger ER stress and to increase eIF2α phosphorylation.

### Role of host autophagy in *T. gondii* proliferative potential

**Figure 5:**
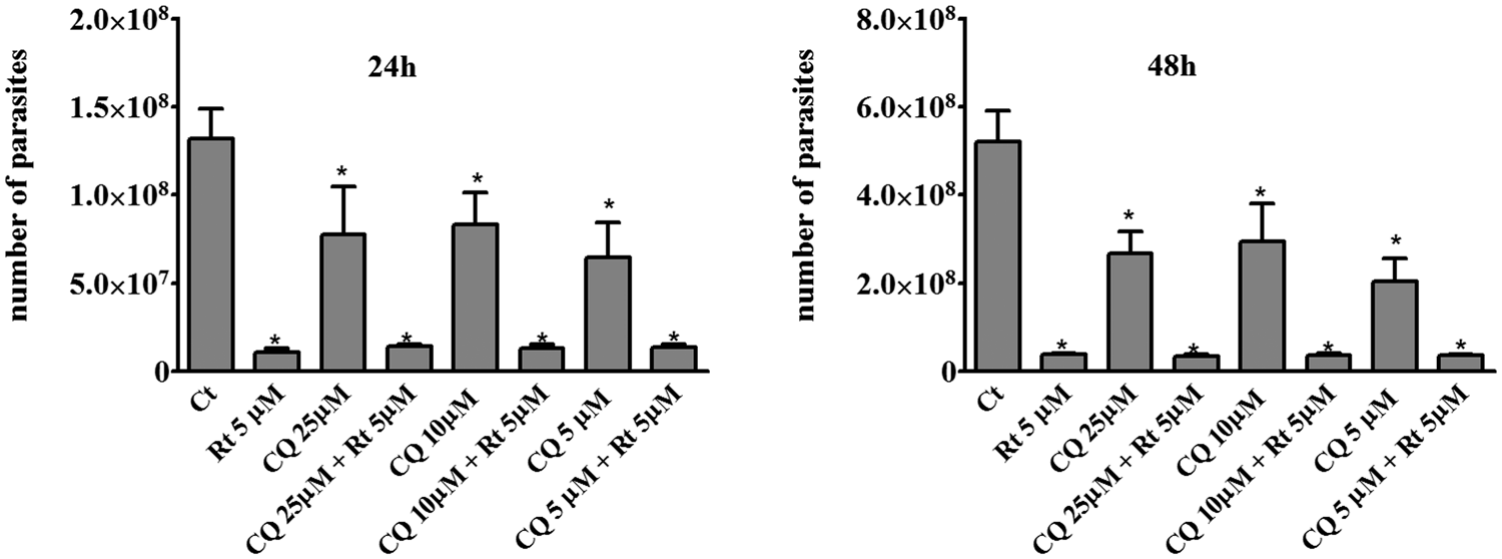
Autophagy is not responsible for the Rottlerin anti parasitic action. BeWo cells were infected with *T. gondii* (host cell: tachyzoites, 1:5) for 3 hours then treated 5 µM Rottlerin plus increasing concentrations of CQ (5, 10, 25 µM). *T. gondii* proliferation was evaluated as described in Fig. 2 after 24 and 48 hours *p < 0.0001. Data were expressed as mean ± SEM of the number of tachyzoites calculated in relation to a reference curve and are representative of three independent experiments performed in quintuplicate.

To evaluate the role of autophagy in the Rottlerin anti-parasitic action, the autophagy inhibitor CQ, which blocks the late acidification steps of the process, was used. As shown in Fig. 5, CQ failed to prevent the Rottlerin inhibition of parasite replication, indicating that, although the autophagic pathway was clearly stimulated, autophagy was not responsible for the Rottlerin protective mechanism against infection. Moreover, in agreement with the notion that *T. gondii* usually exploits the autophagic machinery of the host cell for survival purposes, CQ alone decreased parasite growth inside the host cell.

## Discussion

Up to now, an effective treatment against *T. gondii*, with no toxicity for the host, has not been yet discovered. In fact, most of the current therapies exhibit a high degree of toxicity^8, 31^.

The recommended treatment adopted for toxoplasmosis is the combination of the antibiotics sulfadiazine, pyrimethamine and folinic acid or spiramycin. However, this regimen is commonly associated with many adverse effects, including teratogenic effects in the first trimester of pregnancy^32^. Thus, researchers are encouraged to disclose new, less toxic drugs that actively combat/prevent *T. gondii* infection.

In the current study, BeWo cells were used as an *in vitro* model of human trophoblast. Our group for many years is engaged in the study of congenital toxoplasmosis focusing the research on the placenta^33, 34^. Many *in vitro* models have been established for human placental trophoblast studies, among which cell lines, including BeWo cells, are widely used and particularly advantageous in terms of reproducibility to evaluate the net effect of therapeutic agents on laboratory challenge tests with pathogens^35, 36^. We found that Rottlerin sub-toxic concentrations for host cells are able to combat *T. gondii* infection in BeWo cells significantly and dose dependently.

In agreement with the notion that Rottlerin is an autophagy inducer, we have shown that Rottlerin alone, even at doses not affecting cell viability, can induce autophagy also in BeWo cells. As previously found in MCF-7 cells, also in BeWo cells Rottlerin induced autophagy via mTORC1 inhibition and independently from Beclin-1^5^. Autophagy is a natural defense against parasites and, indeed, we found enhanced cytoplasmic vacuolization and stimulated autophagic flux in infected cells. However, *T. gondii* -induced autophagy was both Beclin-1 and mTORC1-independent, indicating that the two autophagy inducers engage different pathways. Recent studies by Sellek *et al*.^37^ reported that the IFNγ-mediated *T. gondii* control in human cells occurred through a mechanism different from both canonical autophagy (Beclin 1-independent) and xenophagy. Despite the different experimental model, Sellek’s and our results converge on a non-canonical (Beclin 1-independent) autophagic-like pathway triggered by *T. gondii* in host cells.

Our results are also in agreement with the earlier Wang’s study^38^ which demonstrated that *T. gondii*-induced autophagy in host cells was mTOR-independent and somehow dependent on a calcium-sensitive step.

Although further work is needed to identify the exact mechanism of *T*. *gondii*-induced host autophagy, the slight expansion of the endoplasmic reticulum (ER) observed in infected BeWo cells (Fig. 3G–I), pointed towards a possible *T. gondii*-induced ER stress/eIF2 kinase (PERK)-dependent autophagic pathway. Indeed, eIF2 kinases can promote autophagy, via phospho-eIF2α-ATF4, by inducing transcription of a set of autophagy genes^29^.

The association between the parasitophorous vacuoles (PV) and the host ER, which could be a source of membrane for the enlargement of the PV, dates back to 1997^39^ and it has been confirmed in more recent studies showing, by electron microscopy, that PVs were surrounded by ER^40^. It is very possible that this tight interaction could led to ER stress and trigger the so called unfolding protein response in the host. Consistently, recent studies reported that apoptosis of mice trophoblasts and neural stem cells infected with *T. gondii* was triggered by ER stress^41, 42^. Although no (apoptotic) death was observed in infected BeWo cells, the results from Western blotting analysis of cell lysates after infection showed increased eIF2α phosphorylation, indicating the activation of the ER stress-stimulated kinase, PERK. We assumed that this could be the underlying mechanism of *T. gondii*-triggered host autophagy.

Accordingly to the double stimulation (by Rottlerin and infection-induced ER stress and Rottlerin-inhibited mTORC1), we observed an exacerbated autophagic response, both at the microscopic (TEM) and the biochemical (Western blotting of LC3I/II and p62) level, in infected cells after Rottlerin exposure.

However, the over-stimulation of autophagy was not the mechanism by which Rottlerin decreased parasite replication, since the drug had superimposable effects in the absence or in the presence of the autophagy inhibitor CQ. Moreover, consistently with the notion that *T. gondii* usually exploits the autophagic machinery of the host cell for survival purposes, we observed that CQ alone hampered parasite growth inside the host cell. However, given the general ability of CQ to inhibit organelles acidification, we cannot exclude that it could have a direct effect on parasite as well. Moreover, because CQ does not block the formation of autophagosoma, it is possible, in theory, that this could be sufficient to arrest parasite replication even in the absence of a complete autophagic flux. However, because Rottlerin and CQ have not an additive effect, this eventuality can be ruled out. At the same time, the lack of synergism/antagonism between Rottlerin and CQ confirms the irrelevance of autophagy induction in the Rottlerin outcome.

**Figure 6:**
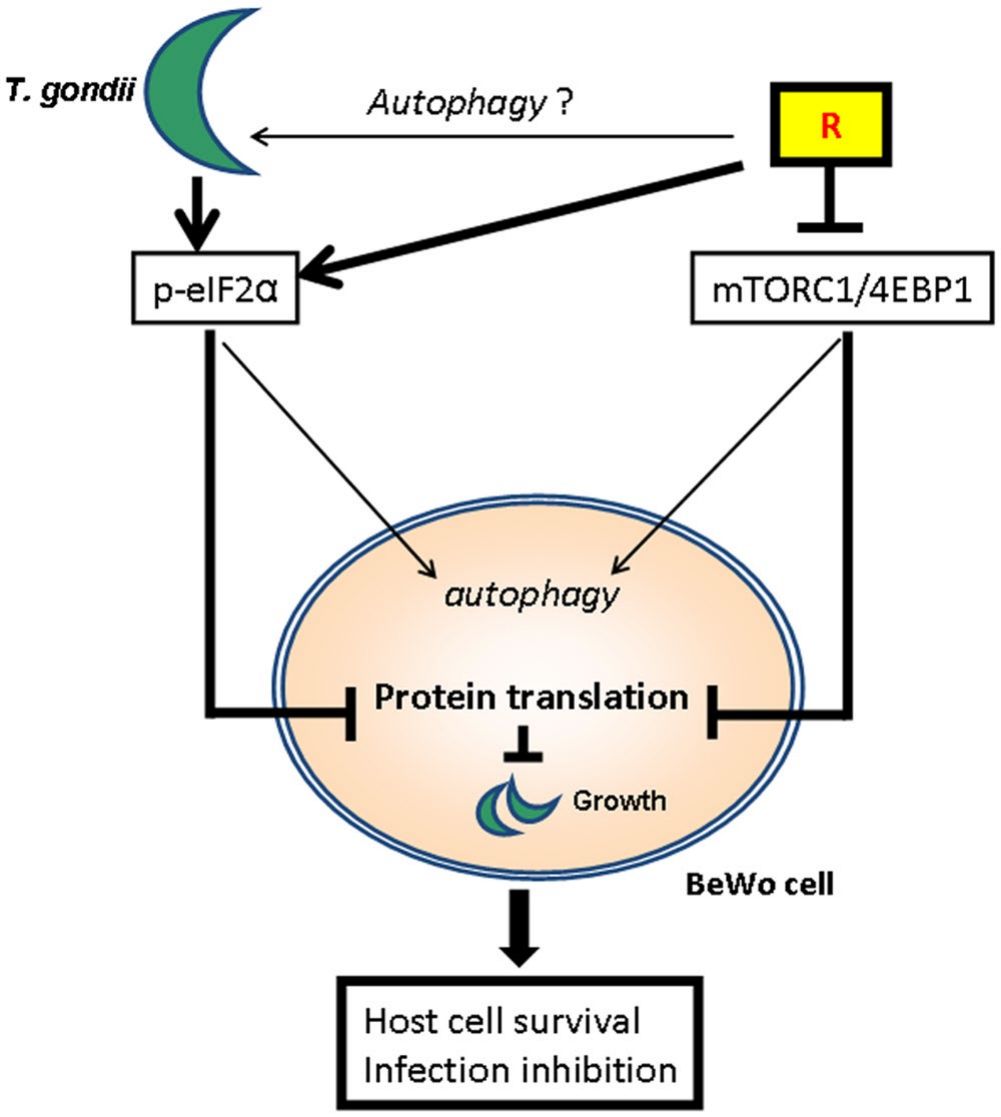
Schematic representation of the Rottlerin action against *Toxoplasma gondii* infection. Rottlerin inhibited the mTORC1/4EBP-1 axis and, synergically with *T. gondii*, induced eIF2α phosphorylation. Both events led to autophagy induction and inhibition of protein synthesis.

Overall, these findings suggest that a novel mechanism, alternative to autophagy, are responsible for the Rottlerin protective effect against *T. gondii* infection in BeWo cells; such a mechanism could be host protein synthesis curtailing (Fig. 6).

Indeed, in addition to mTORC1, which is a regulator of cap-dependent mRNA translation, phosphorylation of eukaryote initiation factor 2a (eIF2a) on serine 51 by ER stress-activated kinases potently inhibits the initiation of protein translation. It is well known that *T. gondii* actively appropriates numerous host cell processes and significant changes in host cell transcripts occur during the course of the infection, indicating that the contribution of host transcriptional/translational activities is essential for successful infection^43^.

The inhibition of protein translation, that inevitably occurs in concomitance with autophagy induction, appeared to be a very effective process in reducing parasites proliferation, especially when synergically promoted by *T. gondii* infection, via ER stress/eIF2α, in combination with Rottlerin, via mTORC1/4EBP.

In closing, the results of the current study encourage the development of Rottlerin-based new therapeutic formulations against *T. gondii* infection for which a limited number of effective and safe drugs are currently available. Particularly, the results here obtained might be useful to combat and/or prevent the possible severe consequences for the fetus by maternal infection during pregnancy.

## Materials and Methods

### Cell culture

The choriocarcinoma-derived BeWo cell line, used as experimental model of trophoblast cells, was obtained from the American Type Culture Collection, (Manassas, VA, USA). Cells were grown in RPMI 1640 medium (Cultilab, Campinas, SP, Brazil) containing 10% fetal bovine serum (FBS) (Cultilab) supplemented with 25 mM HEPES, 2 mM L-glutamine, 100 U/mL penicillin and 100 μg/mL streptomycin (Sigma Chemical Co., St Louis, MO, USA). Cells were routinely cultured in 75 cm^2^ vented flasks in a humidified 5% CO_2_–95% air atmosphere at 37 °C until 70–80% confluence.

### Rottlerin

Rottlerin (Sigma Chemical Co.) was dissolved in dimethyl sulfoxide (DMSO; vehicle) to a concentration of 20 mM (stock solution). Stock solution was freshly diluted in culture medium and concentrations ranging from 0.5–20 µM were used for the treatments.

### Cell viability

Cell viability was assessed by Trypan blue exclusion assay. Briefly, BeWo cells were seeded in 24-well plates at 5 × 104/mL in RPMI 1640 (supplemented as above described) and 10% FBS. The day after, the medium was substituted with fresh RPMI 1640 with 2% of FBS containing increasing concentrations of Rottlerin (0.5, 1, 2, 5, 10 and 20 µM) or vehicle as control. The cultures were incubated for 24 and 48 hours. Cell viability was assessed also after cell infection. Briefly, BeWo cells were infected and treated with Rottlerin (5 µM) for 24 hours as described thereafter. The number of viable cells was evaluated by detaching with trypsin solution (0.05% trypsin–0.02% sodium EDTA) and using the Countess™ automated cell counter (Invitrogen, Carlsbad, CA, USA), which employs the standard Trypan blue technique. Four replicate counts were determined at each time point. The viability was presented as a percentage of control (vehicle-treated cells).

### Parasite

Tachyzoites of *T. gondii* 2F1 strain derived from the RH strain and gently provided by Dr. Vern Carruthers, Medical School of Michigan University (USA) were used in the current study. These parasites, which constitutively express cytoplasmic β-galactosidase, were propagated in BeWo cells maintained in RPMI 1640 medium supplemented with penicillin, streptomycin, and 2% FBS at 37 °C and 5% CO_2_.

Tachyzoites were harvested by scraping off the BeWo monolayer within 3 days of infection, passed through a 26-gauge needle to lyse any remaining intact host cells and finally centrifuged at 720 × *g* for 5 minutes to pellet the parasite. Parasites, suspended in RPMI 1640 medium, were stained with 0.4% Trypan blue and counted in a hemocytometric chamber for infection experiments.

### Infection of BeWo cells by *T. gondii* and treatments

BeWo cells were cultured in 96-well plates (2 × 10^4^ cells/well/200 µL) in RPMI 1640 with 10% FBS at 37 °C and 5% CO_2_ overnight. Next, BeWo cells were infected with *T. gondii* tachyzoites (2F1 strain) at levels of 5 parasites per cell (5:1). After a 3 hour-infection period, the non-adherent parasites were removed along with the supernatant. The infected cells were washed twice with warm medium, the plates were refilled with fresh medium at 2% of FBS containing the treatment (Rottlerin at 0.5, 1, 2 and 5 µM), and incubated for 24 and 48 hours at 37 °C. In a set of experiments, the infected cells were treated with 5 µM Rottlerin plus 5, 10 and 25 µM chloroquine (CQ) (Sigma Aldrich, St. Louis, MO USA).

*T. gondii* intracellular proliferation was evaluated using a colorimetric β-galactosidase assay as previously described^44^. *T. gondii* intracellular proliferation data were expressed as the number of tachyzoites calculated in relation to the standard curve of 2F1 strain tachyzoites ranging from 1 × 10^6^ to 15.625 × 10^3^ parasites.

### Electron Microscopy

BeWo cells, under the various treatments, were pelleted and fixed overnight with 2.5% glutaraldehyde, 2% paraformaldehyde diluted in 0.1 M phosphate buffered saline (PBS), pH 7.2, washed three times with PBS and post-fixed for 1 hour in 1% OsO4, dehydrated in increasing concentrations of ethanol and embedded in Epon (Sigma and Co.). Ultrathin sections were stained with uranyl acetate and lead citrate and finally examined in a Zeiss EM 109 transmission electron microscope (Zeiss, Jena, Germany).

### Western blotting

After treatments, BeWo were washed with PBS and then cryolysed (three freeze-thaw cycles) with RIPA [50 mM Tris-HCl, 150 mM NaCl, 1% Triton X-100, 1% (w/v) sodium deoxycholate, and 0.1% (w/v) sodium dodecyl sulfate (SDS), pH 7.5] containing a cocktail of protease and phosphatase inhibitors. Cell debris was pelleted by centrifugation at 14.000 × *g* for 15 min. The supernatants were transferred to new tubes, and the protein concentration was measured using the Bradford assay.

Equal amounts of protein samples were resolved on 10%, SDS–polyacrylamide gels under reducing condition. Proteins were electro-transferred onto PVDF membranes and then blocked in 4% non-fat dry milk in Tris-buffered saline pH 7.2 (TBS) containing 0.1% Tween 20 for 2 hours at room temperature. Then, the blots were probed with polyclonal antibodies anti human LC3I/II, p4EBP-1, Beclin 1, peIF2α and PARP (Cell Signaling Technology, Boston, MA USA), SQSTM1/p62 and β-actin (Santa Cruz Biotechnology; Santa Cruz, CA) at dilution of 1:1000 overnight at 4 °C. After washing, appropriate horseradish peroxidase-conjugated IgG was added for 2 hours at room temperature. The blots were then developed by the ECL Detection Reagents and then exposed on photographic film, according to the manufacturer’s instructions or digitalized with CHEMI DOC Quantity One program (BioRad Microscience).

### Statistical analysis

All experiments were repeated at least three times. The data were analyzed with GraphPad Prism 4 using one-way ANOVA and the Bonferroni multiple comparison post-test or Student’s t-test when appropriate. p < 0.05 was considered to be statistically significant.

## Electronic supplementary material

Infection of BeWo cells by T. gondii and treatments
