# Supplementary material for: Rottlerin-mediated inhibition of Toxoplasma gondii growth in BeWo trophoblast-like cells
Source: Sci Rep. 2017 Apr 28;7:1279. doi: 10.1038/s41598-017-01525-6 (PMC5430667; doi:10.1038/s41598-017-01525-6)
Supplement: Supplementary file 1 — Infection of BeWo cells by T. gondii and treatments [file 41598_2017_1525_MOESM1_ESM.doc]

**Supplementary data**

**Rottlerin-mediated inhibition of *Toxoplasma gondii* growth in BeWo trophoblast-like cells.** Francesca Ietta^a^,*, Emanuela Maioli^a^, Elena Daveri^a^, Juliana Gonzaga Oliveira^b^, Rafaela José da Silva^b^, Roberta Romagnoli^a^, Laura Cresti^a^ , Anna Maria Avanzati^a^, Luana Paulesu^a^, Bellisa de Freitas Barbosa^b^, Angelica de Oliveira Gomes^b^, José Roberto Mineo^c^ and Eloisa Amália Vieira Ferro^b^.

^a^Department of Life Sciences, University of Siena, Via A. Moro 4, 53100 Siena Italy;

^b^Laboratory of Immunophysiology of Reproduction, Institute of Biomedical Sciences, Federal University of Uberlândia, Av. Pará 1720, 38405320 Uberlândia, Brazil;

^c^Laboratory of Immunoparasitology, Institute of Biomedical Sciences, Federal University of Uberlândia, Av. Pará 1720, 38405320 Uberlândia, Brazil.

***Infection of BeWo cells by T. gondii and treatments.*** BeWo cells were cultured on 13-mm round glass coverslips into 24-well plates (5 × 10^4^ cells/200 μL/well) in RPMI 1640 with 10% FBS at 37 °C and 5% CO_2_ overnight. Next, BeWo cells were infected with *T. gondii* tachyzoites (2F1 strain) at levels of 5 parasites per cell (5:1). After a 3 hour-infection period, the non-adherent parasites were removed along with the supernatant. The infected cells were washed twice with warm medium, the plates were refilled with fresh medium at 2% of FBS containing the treatment (Rottlerin at 0.5, 1, 2 and 5 µM), and incubated for 24 and 48 hours at 37°C. After the treatments cells were fixed in 10% phosphate-buffered formalin for 2 h and stained with 1% toluidine blue (Sigma Chemical Co.) for 3 s. Coverslips were mounted on glass slides and cells were examined by direct counting under a light microscope for infection index (percentage of infected cells per 200 examined cells) and for number of parasites per cell ^1^.

1. Barbosa, B.F., et al., *BeWo trophoblast cell susceptibility to Toxoplasma gondii is increased by interferon-gamma, interleukin-10 and transforming growth factor-beta1.* Clin Exp Immunol, 2008. **151**(3): p. 536-45.

**Supplementary Figure S1.**


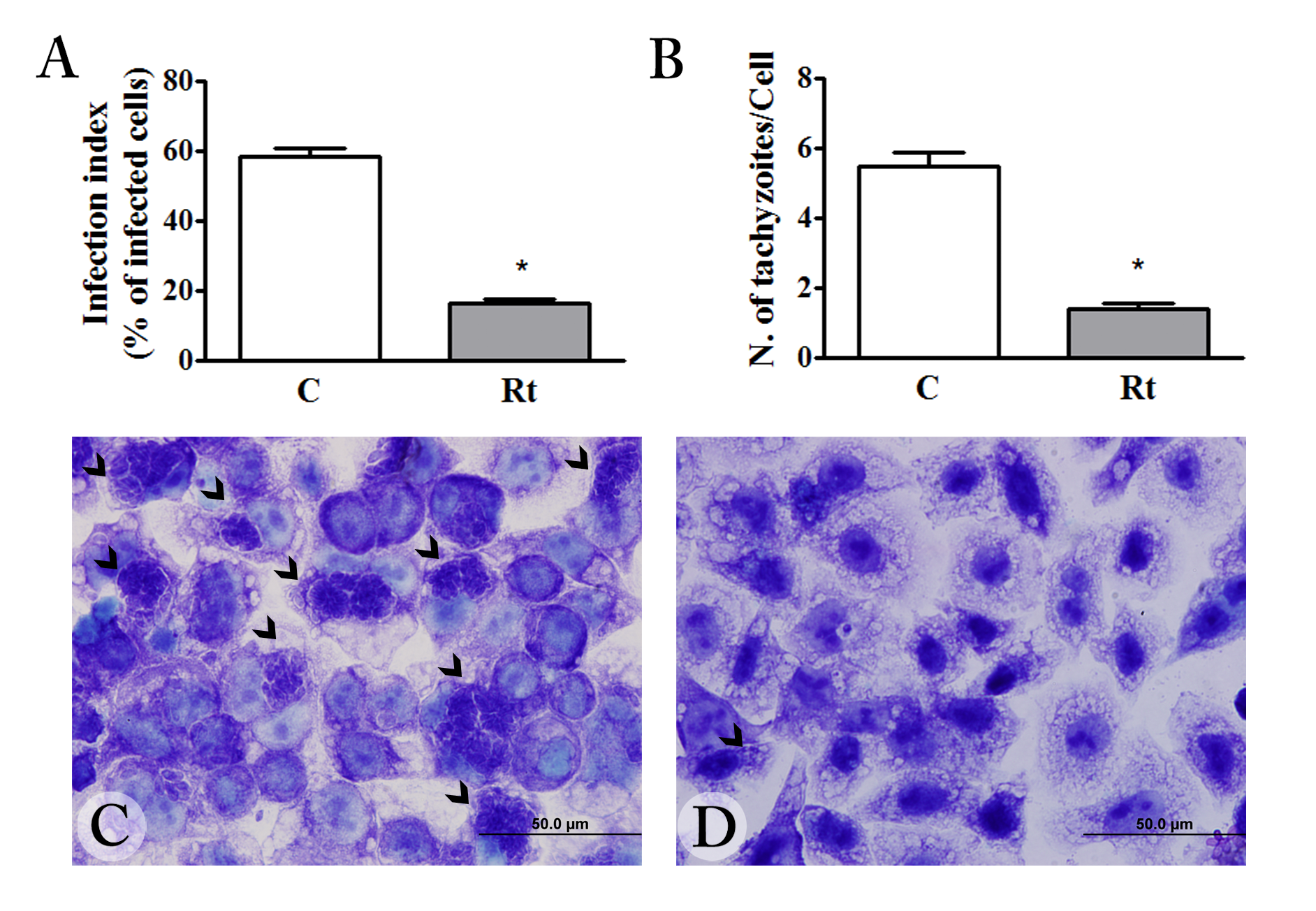


**Figure 1S** **Effect of Rottlerin treatment on *T. gondii.*** Effect of the treatment with 5µM Rottlerin on Toxoplasma gondii index of infection (%) (**A**) and replication (number of parasites per cell) (**B**) in BeWo cells. Representative photomicrograph of BeWo cells infected with of T. gondii **(C)** and infected with *T. gondii* and treated with 5µM Rottlerin **(D)**. Arrow heads indicate parasites inside the parasitophorous vacuoles. Toluidine blue staining; Bars: 50μm.
